# Effect of a Dual PPAR α/γ agonist on Insulin Sensitivity in Patients of Type 2 Diabetes with Hypertriglyceridemia- Randomized double-blind placebo-controlled trial

**DOI:** 10.1038/s41598-019-55466-3

**Published:** 2019-12-12

**Authors:** Nimisha Jain, Shobhit Bhansali, Anura V. Kurpad, Meredith Hawkins, Akhilesh Sharma, Sandeep Kaur, Ashu Rastogi, Anil Bhansali

**Affiliations:** 1 Department of Endocrinology, Post Graduate Institute of Medical Education and Research, Chandigarh, 160012 India; 2 Diabetes Research and Training Center and Division of Endocrinology, Department of Medicine, Albert Einstein College of Medicine, Bronx, New York, USA; 3 Department of Physiology, St. John’s Medical College, Bengaluru, India; 4 Department of Psychiatry, Post Graduate Institute of Medical Education and Research, Chandigarh, 160012 India

**Keywords:** Endocrinology, Endocrine system and metabolic diseases

## Abstract

Saroglitazar is a dual PPAR-α/γ agonist approved for the treatment of diabetic dyslipidemia. In addition to reduction in atherogenic lipids, it may also contribute to improvement in insulin sensitivity through PPAR-α/γ agonism, which remains unexplored. We conducted a randomized, double-blind, placebo-controlled trial in treatment-naive T2DM individuals with serum triglyceride >150 mg/dL. Participants were randomized to receive either saroglitazar 4 mg or placebo (1:1) daily for 4 months (n = 30). Insulin sensitivity (SI_clamp_) was studied using hyperinsulinemic-euglycemic clamp at baseline and at 4 months. We observed a significant reduction in TG (p = 0.001), HbA1c (p = 0.019) and fasting plasma glucose (p = 0.019) and significant increase in HDL-C levels (p < 0.01) with saroglitazar compared to placebo. Further, patients on saroglitazar had a greater improvement in SI_clamp_ (p = 0.026) with the effect persisting despite adjusting for baseline weight, TG, HDL-C and HbA1c (p = 0.002). This was accompanied with significant increase in HOMA-β (p = 0.01) in the saroglitazar group and change in HOMA-β showed a trend towards significance with SI_clamp_ (r = 0.503, p = 0.056). However, change in SI_clamp_ did not significantly correlate with reduction in HbA1c and TG. We conclude that saroglitazar effectively reduces hypertriglyceridemia and improves insulin sensitivity along with β-cell function by reduction in gluco-lipotoxicity and possibly directly through PPAR-γ agonism in patients ofT2DM with hypertriglyceridemia.

## Introduction

Diabetic dyslipidemia, also known as atherogenic dyslipidemia comprises a triad of “raised triglycerides^1^, higher proportion of small dense low density lipoprotein-cholesterol (sdLDL-C) and low high density lipoprotein cholesterol-(HDL-C)^2^”. The presence of small dense LDL-C particles lead to accelerated atherosclerosis resulting in increased cardiovascular (CV) morbidity and mortality^3^. Statins, fibrates and omega-3 fatty acids are used for the management of diabetic dyslipidemia. While statins reduce cardiovascular events and decrease mortality, considerable residual cardiovascular risk persists despite receiving statin therapy^4^. Several studies designed to target this residual CV risk have shown variable outcomes. The AIM-HIGH (Atherothrombosis Intervention in Metabolic Syndrome With Low HDL/High Triglycerides)^5^ and HPS2-THRIVE (Heart Protection Study 2-Treatment of HDL to Reduce the Incidence of Vascular Events)trials^6^ aimed at raising HDL-C with niacin but failed to demonstrate the reduction in CV mortality.

Hypertriglyceridemia increases CV risk by resulting in remodelling of HDL-C and LDL-C particles rendering them smaller and denser. sdLDL-C particles are more atherogenic and sd HDL-C particles are dysfunctional, therefore increasing the CV disease risk^7^. A rise in TG levels by 1 mmol/l is estimated to raise the CV risk by 32% and 76% in men and women, respectively^8,9^. In addition, a global case-control study showed that microvascular disease odds ratio increased by a factor of 1.16 (95% confidence interval, 1.11–1.22) for every 0.5 mmol/L increase in triglycerides^10^, which make increased TGs a worthy target for treatment.

Fibrates through their action on PPAR-α are the “traditional” drug of choice to target hypertriglyceridemia. However, initial enthusiasm was dampened mainly owing to the results of two studies: the Fenofibrate Intervention and Event Lowering in Diabetes (FIELD) study^11^ and the Action to Control Cardiovascular Risk in Diabetes (ACCORD) study^12^. Both these studies could not demonstrate a significant reduction in CV risk either alone^11^ or in combination with statins^12^.

Glitazars are a group of drugs with dual PPAR- α/γ agonist action. A number of glitazars were developed, however, they could not make their position in the management of dyslipidemia owing to unacceptable adverse effects such as peripheral edema (faglitazar)^13^, carcinogenic potential (ragaglitazar)^14^, cardiovascular side-effects (muraglitazar)^15^ and bone marrow and renal toxicity (tesaglitazar).

Saroglitazar is a novel PPAR-α/γ agonist, recently approved for diabetic dyslipidemia in India. As PPAR-α are predominantly expressed in the liver, it effectively reduces circulating atherogenic lipids and consequent decrease in lipotoxicity. Saroglitazar also reduces HbA1c partly through reduction in lipotoxicity as well as through its moderate PPAR-γ agonistic activity. A previous study showed that saroglitazar as an add-on to metformin had a greater lowering of TG and HbA1c, as compared to fenofibrate^16^. However, its efficacy in improving insulin sensitivity has been studied only in animal models till now^10^ and remains unexplored in human subjects. Therefore, we planned to study the effect of saroglitazar on insulin sensitivity in patients with type 2 diabetes mellitus (T2DM) with hypertriglyceridemia by hyperinsulinemic-euglycemic clamp.

## Methods

### Study design

It was designed as a double-blind placebo controlled randomised trial. The trial was registered at http://ctri.nic.in on 31 October 2017 (CTRI/2017/10/010306). The study was approved by the Institutional Ethics Committee of Post Graduate Institute of Medical Education and Research, Chandigarh. Written signed informed consent was obtained from all the patients included in the study. Participants were closely monitored to detect and promptly manage adverse events. The study was conducted following the principles of Declaration of Helsinki and Good clinical Practice as laid down by Indian Council of Medical Research^17^.

### Patients

Treatment-naïve T2DM patients were recruited from the outpatient clinic at a tertiary care referral centre in India. Patients aged between 30 and 60 years, with disease duration <5 years, GAD-65 antibody negative, HbA1c 7.0–9.0%, serum fasting triglyceride >150 mg/dL and BMI 23–35 Kg/m^2^ were included.

Patients with type 1 diabetes mellitus or secondary diabetes, past history of diabetic ketoacidosis or having ketonemia or ketonuria, uncontrolled hypertension, thyroid disorder, renal dysfunction [defined by estimated glomerular filtration rate (eGFR) <60 ml/min/m^2^], hepatic dysfunction (serum aspartate transaminase/alanine transaminase (AST/ALT) ratio more than 2.5 times upper limit of normal, and/or total serum bilirubin more than 2 times upper limit of normal), myopathies, receiving statins, fibrates, hormone replacement therapies or steroids, seropositive for human immunodeficiency virus, hepatitis C virus or hepatitis B virus infection, recent cardiovascular event (<6 months), history of malignancy, active infection and alcohol (>14 units/week or 112 gm pure alcohol for men, >7 units/week or 56 gm pure alcohol for women)^18^ or drug abuse were excluded from the study. An informed consent was obtained from every patient prior to inclusion in the study. Since this was a pilot study, with 90% power and two-sided 5% significance for standardised medium effect size of 0.5, a sample size of 30 patients was considered optimal for the study.

After screening, subjects were randomised to receive saroglitazar 4 mg and placebo in 1:1 distribution for 4 months. Life-style modification was insisted to all patients as per ADA recommendations^19^. Patients were advised to perform self-monitoring of blood glucose (SMBG) including a five-point glucose profile once a week. Compliance was ensured by weekly phone call by the investigator. Tablet glimeperide (1 to 2 mg) was used as a ‘rescue therapy’ in either group, when fasting plasma glucose (FPG) exceeded 130 mg/dl or post prandial plasma glucose (PPG) >180 mg/dl. Block randomization was done and random numbers were generated^12^ using Microsoft Excel. Randomization concealment was achieved by placing random number codes in sealed tamper- proof envelopes and blinding was not disclosed to either the trial participant or assessor (NJ). NJ enrolled participants and AB allocated them to intervention.

### Study assessments

Five visits were planned, including first visit at baseline and then monthly till the completion of the study. Liver function tests, renal function tests, lipid profile and fasting and 2 hour post- prandial glucose were performed at each visit. At baseline and at the end of 4 months, anthropometry was performed and glycated haemoglobin (HbA_1c_), C -peptide and high sensitivity C-reactive protein (hsCRP) were measured. HbA1c was measured using automated high-performance liquid chromatography (HPLC)-based system using ion-exchange cartridge (D-10, Bio-Rad Laboratories, Inc., Hercules, CA, USA). C-peptide estimation was done at baseline and 4 months by electrochemiluminiscence immunoassay (ECLIA) (Elecsys 2010, Roche Diagnostics GmbH, Mannheim, Germany). Body composition was also measured by dual energy x-ray absorptiometry (Hologic Inc, USA) at baseline and 4 months.

Patients underwent hyperinsulinemic euglycemic clamp study at inclusion and at the end of 4 months as per DeFronzo’s clamp technique^20^. Subjects were requested to refrain from vigorous exercise prior to the procedure and were asked to report at 0630 h after an overnight fast of 10 hours. Clamp was started at FPG levels of 110 mg/dl. If patient’s plasma glucose was more than 140 mg/dl, low dose insulinization procedure was undertaken^21^. A priming insulin infusion was given for initial 10 minutes, followed by a constant infusion for the next 110 minutes at the rate of 40 mU/m^2^ to achieve plasma insulin concentration of 100 µU/ml. At the 4^th^ minute, glucose infusion (25% dextrose) was started intravenously via infusion pump, with rate set at 2.0 mg/kg/min up to 10^th^ min. subsequent infusion rate was adjusted depending on the arterialized plasma glucose values obtained every 5 minutes with aim of maintaining plasma glucose concentration at an average value of 90 mg/dl for next 110 min. The blood samples for glucose measurements were drawn into ethylene diamine tetra acetate (EDTA) tubes and the separated plasma was analyzed by the glucose oxidase method on a bedside glucose analyzer (GM9D, Analox instruments, London, UK). For insulin levels measurement, sample was collected every 20 min in heparinized tubes and plasma stored at −80 °C until analysis, which was done by ECLIA (Elecsys 2010, Roche Diagnostics, GmbH, Mannheim, Germany). Glucose disposal rate and insulin sensitivity (SI) were calculated over 40 to 120 min of the clamp. Mean steady state plasma glucose values, glucose infusion rates and insulin values were obtained during the study.

Glucose infusion rate = glucose disposal rate (M)

Insulin sensitivity index (SI_clamp_): = M/(G × ΔI) where M is glucose disposal rate and ΔI is the difference between fasting and steady-state plasma insulin concentrations^22^.

HOMA-IR = [fasting plasma insulin (μU/ml) × fasting plasma glucose (mmol/L)]/22.5^23,24^

HOMA-β = 20 × fasting plasma insulin (μU/ml)/[fasting plasma glucose (mmol/L)−3.5]^23^

QUICKI = 1/[log(fasting insulin in μU/ml) + log(fasting glucose in mmol/L)]^25^.

### End points

Primary end point was the change in insulin sensitivity (SI_clamp_) and glucose metabolism (M) by hyperinsulinemic-euglycemic clamp and HOMA-β at 4 months with saroglitazar as compared to placebo. Secondary end points included change in body weight, fasting plasma glucose, post-prandial plasma glucose, HbA1c, fasting lipid profile, insulin and C peptide levels.

### Statistical analysis

Data obtained was analysed using Statistical Package of Social Sciences (SPSS) version 20. (Armonk, NY: IBM Corp). Results obtained were expressed as mean value ± 2 standard deviation (parametric data) or as median value and IQR (non-parametric data). Data was analysed using intention to treat^26^ analysis. Missing data was handled with last observation carried forward (LOCF). Student’s t-test (parametric data), Mann Whitney U test (non-parametric data) and Wilcoxon signed rank test (non-parametric data) were used for comparisons between saroglitazar and placebo groups. Correlation analysis was carried out using Pearson’s correlation and Spearman’s rho as appropriate. Analysis of covariance (ANCOVA) was used to study the effect of treatment on between group differences with treatment given (placebo or saroglitazar) as fixed factor, change in parameter as dependent variable and baseline value of the parameter being assessed as covariate.

### Ethics approval and consent to participate

The study was performed according to the declaration of Helsinki and was approved by the Institutional Ethics Committee of Postgraduate Institute of Medical Education and Research, Chandigarh, India. Written informed consent was obtained from all the patients to participate in the study.

## Results

### Demographic characteristics

**Figure 1:**
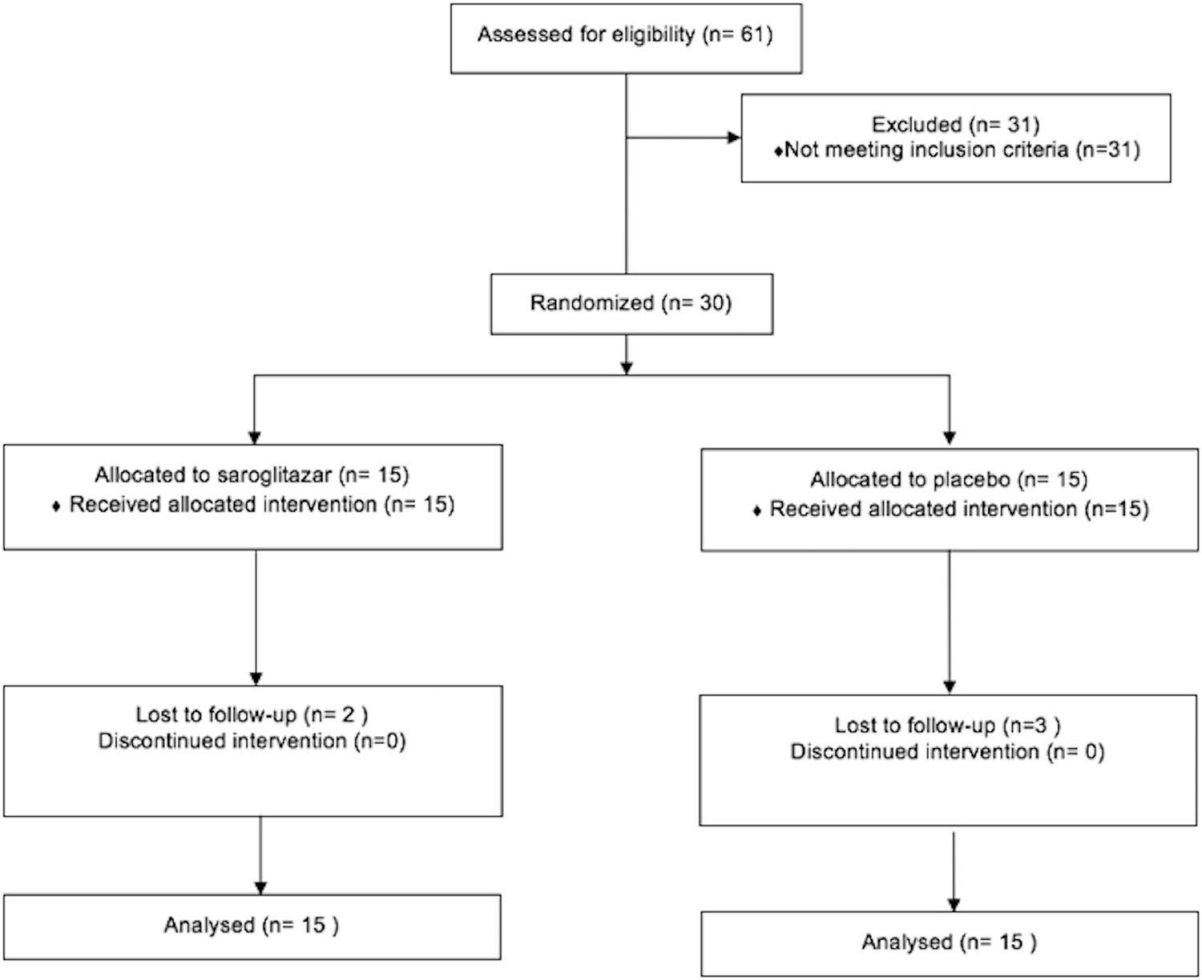
Flowchart showing patient randomisation and disposition.

A total of 61 patients were screened from January, 2017 till June, 2018. Among them 30 had fulfilled the inclusion criteria and were recruited in the study (Fig. 1). Five patients were lost to follow up as they withdrew the consent (3 placebo, 2 saroglitazar), all within the first month of inclusion. The mean age of participants was 44 ± 9.5 years (27 men). The median duration of diabetes in saroglitazar group was 3 months (1–12 months) and in placebo group was 2 months (1–6 months). The mean weight, BMI, waist circumference and percentage body fat were comparable between the two groups (Table 1).The dose of glimepiride at the completion of the study was comparable in either group (2 mg).

**Table 1:** Showing baseline parameters and changes in lipid profile between saroglitazar and placebo groups at baseline and 4 months.

Parameter	Placebo (n=15)	Saroglitazar (n=15)	P value
Age (years)	47 ± 8.8	40.9 ± 9.6	0.083
Sex (M:F)	12:3	15:0	0.068
Duration of diabetes (months)	2 (1–12)	3 (1–6)	0.412
Height (cm)	164.5 ± 8.1	169.7 ± 5.6	0.051
**Weight (kg)**			
Baseline	75.6 ± 11.0	78.7 ± 9.8	0.418
At 4 months	76.1 ± 11.2	79 ± 10.4	
Change	0.4 ± 2.1	0.2 ± 2.2	0.782
**Body Mass Index (kg/m²)**			
Baseline	27.8 ± 2.5	27.3 ± 2.5	0.389*
At 4 months	27.9 ± 2.8	27.3 ± 2.8	
Change	0.14 ± 0.8	0.05 ± 0.8	0.967*
**Waist Circumference (cm)**			
Baseline	97.2 ± 6.5	97.6 ± 6.9	0.967*
At 4 months	98.2 ± 7.3	97.3 ± 6	
Change	1 ± 2.4	−0.2 ± 2.4	0.389*
**Body Fat (%)**			
Baseline	27.7 ± 3.4	27.2 ± 2.3	0.210
At 4 months	27.7 ± 2.7	27.3 ± 2.6	
Change	−0.03 ± 1.3	0.05 ± 0.8	0.829
**Total Cholesterol (mg/dl)**			
Baseline	217.6 ± 45.5	192.4 ± 42.9	0.129
At 4 months	189.2 ± 49.9	178.9 ± 40.2	
Change	−28.4 ± 44.3	−13.4 ± 32.9	0.303
**Triglycerides(mg/dl)**			
Baseline	236.3 ± 83.1	325.6 ± 129.3	0.019*
At 4 months	245.5 ± 109.1	209.4 ± 124.4	
Change	9.2 ± 82.5	−116.2 ± 85.9^a^	0.001*
**High Density Lipoprotein- Cholesterol (mg/dl)**			
Baseline	45.3 ± 8.5	37.49 ± 9.6	0.026
At 4 months	41 ± 7.9	42.95 ± 10.6	
Change	−4.25 ± 6	5.46 ± 4.6^a^	<0.01
**Low Density Lipoprotein-Cholesterol (mg/dl)**			
Baseline	146.7 ± 45.3	116.4 ± 36.3	0.053
At 4 months	119.4 ± 44.2	112.6 ± 31	
Change	−27.2 ± 49.8	−3.7 ± 25.8	0.119
**Apolipoprotein B (mg/dl)**			
Baseline	133.5 ± 30.7	114.4 ± 24.3	0.070
At 4 months	115.7 ± 33.8	103.6 ± 26.9	
Change	−17.8 ± 32.8	−10.8 ± 24.2	0.512
**High sensitivity C-Reactive Protein (mg/l)**			
Baseline	4.3 (0.7–19.3)	1.6 (0.5–6.7)	0.089
At 4 months	3.7 (1.2–11.7)	1.9 (0.48–6.7)	
Change	0 (−0.9–0.5)	−0.01 (−0.87–0.47)	0.870
Median glimepiride dose (mg)	2 (1–2)	2 (1–2)	0.769

^a^Significant change (p < 0.05) within the group from baseline.

*Mann Whitney U.

### Baseline metabolic parameters

Saroglitazar arm had significantly higher serum triglycerides (p = 0.019) and lower HDL-C levels (p = 0.026) as compared to the placebo arm (Table 1), whereas total cholesterol, low density lipoproteins (LDL-C) and apolipoprotein B were comparable in both the groups at baseline. The two groups had similar levels of hsCRP, fasting and post prandial plasma glucose, HbA1c, fasting plasma C-peptide and fasting plasma insulin. Hyperinsulinemic euglycemic clamp study revealed that the groups were similar with respect to glucose metabolism (M) and insulin sensitivity (SI _clamp_) as well as HOMA-IR, log HOMA, HOMA-β and 1/HOMA. (Table 2).

**Table 2:** Showing changes in glycemic parameters between saroglitazar and placebo groups at baseline and 4 months.

Parameter	Placebo (n=15)	Saroglitazar (n=15)	P value
**Fasting plasma glucose (mg/dl)**			
Baseline	141.3 ± 16.2	154.9 ± 23.7	0.078
At 4 months	132 ± 24.2	115.8 ± 28.2	
Change	−9.9 ± 20.2	−39.1 ± 39.2^a^	0.019
**Post prandial plasma glucose (mg/dl)**			
Baseline	239.4 ± 41.6	240.73 ± 40.1	0.929
At 4 months	214.4 ± 35.8	189.4 ± 47.5	
Change	−25 ± 32.9	−51.3 ± 42.9^a^	0.070
**Fasting plasma Insulin**			
Baseline	15.9 (11.9–18.1)	10.4 (9.5–17.7)	0.161
At 4 months	17.1 (14.2–18.5)	12.2 (11.2–17.7)	
Change	1.7 (0–4.5)	1.8 (−1.73–2.7)	0.624
**Fasting plasma C-peptide**			
Baseline	2.8 ± 0.7	2.6 ± 0.8	0.550
At 4 months	3.24 ± 0.96	2.6 ± 0.7	
Change	0.4 ± 1.2	−0.005 ± 0.6	0.234
HbA1c (%)			
Baseline	7.7 ± 0.6	8 ± 0.7	0.235
At 4 months	7.2 ± 1	6.7 ± 1	
Change	−0.5 ± 0.7	−1.34 ± 1^a^	0.019
**Glucose metabolism (M)(mg/kg.min)**			
Baseline	1.5 ± 1.2	1.6 ± 0.9	0.539*
At 4 months	1.4 ± 0.9	3.2 ± 2.3	
Change	−0.15 ± 0.99	1.4 ± 2.7^a^	0.126*
**Insulin sensitivity (M/I) [100 X (mg/kg) per μU/ml]**			
Baseline	2.3 (1.42–4.47)	2.9 (1.3–6.6)	0.775
At 4 months	3.0 (2.1–3.8)	6.1 (2.09–20.03)	
Change	0 (−0.21–0.9)	0.8 (0–13.14)^a^	0.026
**HOMA-IR**			
Baseline	4.8(4.4–6.6)	4.3(3.72–6.6)	0.267
At 4 months	5.2 (2.2–15.5)	3.1 (2.65–6.6)	
Change	0 (−0.11–1.8)	−0.8 (−1.91–0)	0.081
**HOMA-β**			
Baseline	72.3 ± 24.87	56.7 ± 31.46	0.143
At 4 months	88.1 ± 24.89	110.7 ± 44.94	
Change	12.4 ± 38.51	54.1 ± 44.0^a^	0.01
**Log** **HOMA**			
Baseline	1.6 ± 0.31	1.5 ± 0.49	0.508
At 4 months	1.6 ± 0.58	1.3 ± 0.6	
Change	−0.03 ± 0.56	−0.3 ± 0.4^a^	0.208
**1/HOMA**			
Baseline	0.2 ± 0.07	0.2 ± 0.11	0.318
At 4 months	0.2 ± 0.105	0.30 ± 0.16	
Change	0.1 ± 0.09	0.06 ± 0.16	0.302
**QUICKI**			
Baseline	0.3 ± 0.01	0.3 ± 0.02	0.369
At 4 months	0.3 ± 0.02	0.3 ± 0.02	
Change	0 ± 0.01	0.009 ± 0.02	0.232

^a^Significant change (p < 0.05) within the group from baseline.

*Mann Whitney U.

### Study end points

#### Primary end points

**Figure 2:**
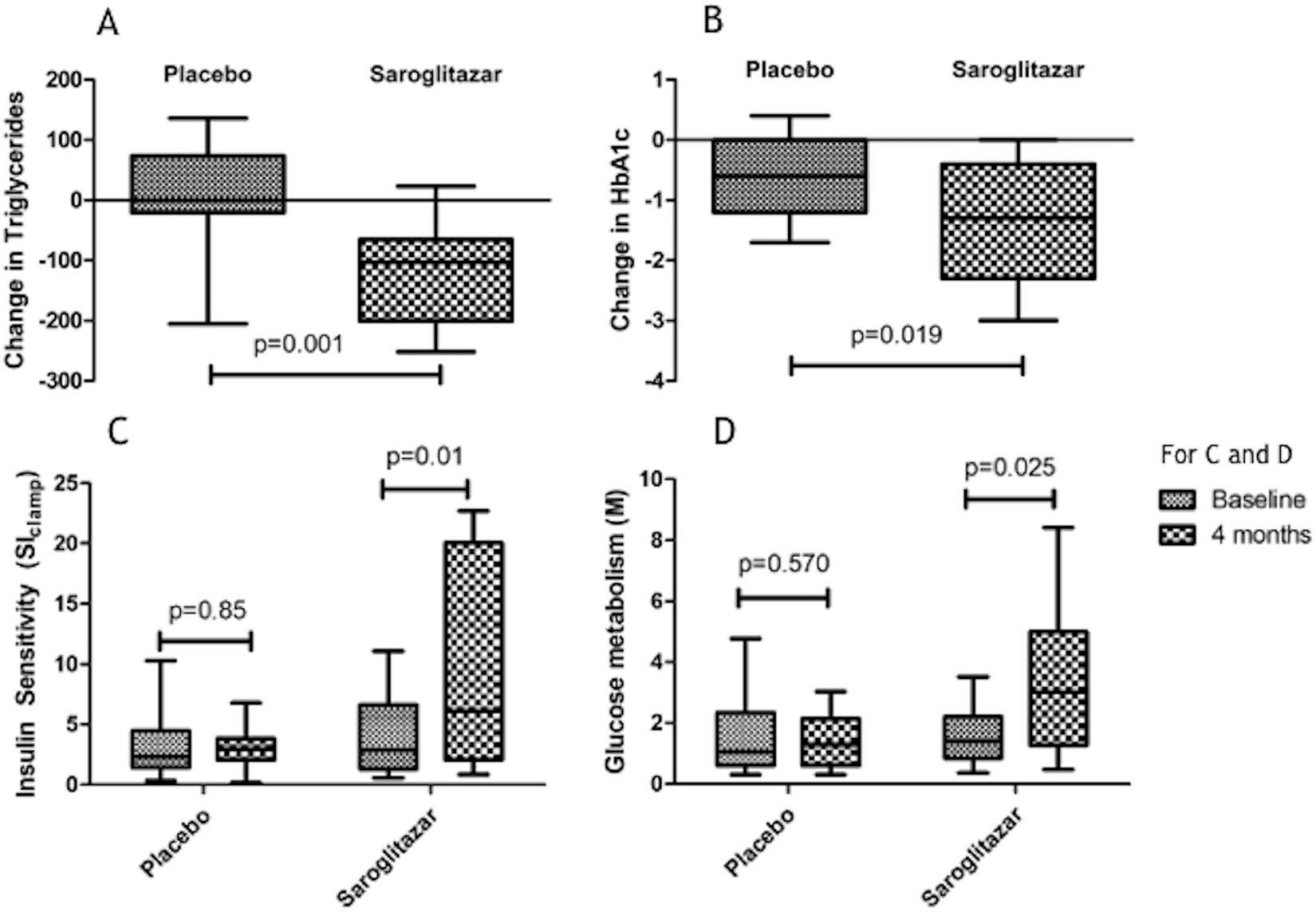
showing changes in triglyceride, HbA1c, insulin sensitivity (SI_clamp_) and glucose metabolism (M) between saroglitazar and placebo groups.

After 4 months of the study period, saroglitazar improved glucose metabolism (M) (P = 0.025) and SI_clamp_(p = 0.011) significantly, as compared to the baseline levels (Fig. 2).Further, SI _clamp_ improved to a greater extent in saroglitazar group as compared to placebo group {2.9 (1.33–6.64) to 6.1 (2.09–20.03) vs 2.3 (1.42–4.47) to 3.0 (2.1–3.8), p = 0.026, all values in [100 × (mg/kg) per μU/ml]}. The effect of saroglitazar on SI_clamp_ at 4 months persisted despite controlling for baseline weight, TG, HDL-C and HbA1c (partial η 2 = 0.342, p = 0.002). However, change in SI_clamp_ did not correlate with change in TG (r = −0.198, p = 0.479) and HbA1c levels (r = −0.415. p = 0.124). HOMA- β increased significantly (p = 0.01) in the saroglitazar group as compared to placebo and inversely correlated with change in fasting plasma glucose (r = 0.749, p = 0.001). However, change in HOMA-β did not significantly correlate with changes in TG (r = −0.098, p = 0.729) and HbA1c levels (r = −0.488, p = 0.065). Nonetheless, change in HOMA-β showed a trend towards significance with SIclamp (r = 0.503, p = 0.056).

#### Secondary end points

Patients in saroglitazar arm had a greater improvement in HbA1c (−1.34 ± 1 versus −0.5 ± 0.7 mg/dl, p = 0.019) and fasting plasma glucose (p = 0.019) as compared to placebo. Saroglitazar also resulted in higher fall in TG levels (p = 0.001) and the significant rise in HDL-C levels (p < 0.01) even after correcting for baseline inequalities (Fig. 2). Further, changes in fasting plasma insulin (p = 0.624), C-peptide (p = 0.234), body weight (p = 0.782),HOMA-IR (p = 0.081), log HOMA (p = 0.208), 1/HOMA (p = 0.302) and QUICKI (p = 0.232) were not significantly different between the two groups (Table 2).

#### Safety profile

No episodes of hypoglycemia, were noted during the clamp studies. No adverse effects including myalgias, peripheral edema, weight gain, hypoglycemia, transaminitis and renal dysfunction were noted in either group during the study period.

## Discussion

This is the first study to evaluate the effects of saroglitazar, a dual PPAR-α/γ agonist, on insulin sensitivity by hyperinsulinemic euglycemic clamp in patients of T2DM with hypertriglyceridemia. Our study showed that saroglitazar treatment resulted in significant reduction in triglycerides, fasting plasma glucose and HbA1c along with increase in HDL-C levels. Further, a significant improvement in whole body insulin sensitivity was observed in the saroglitazar group as compared to placebo. This was accompanied with increase in HOMA-β and a change in HOMA-β showed a trend towards significance with improvement in insulin sensitivity. However, improvement in insulin sensitivity and β-cell function did not correlate either with decrease in HbA1c or with decline in triglycerides.

Insulin resistance is the key abnormality in the pathophysiology of T2DM accompanied with progressive decline in β-cell function, resulting in emergence of hyperglycemia^27^. Various modalities have been employed to estimate the insulin resistance including HOMA-IR,QUICKI and Matsuda index; however, the ‘gold standard’ remains the hyperinsulinemic-euglycemic clamp^28^. HOMA-IR measurement is useful in healthy and prediabetes individuals as well for epidemiological studies; however, with the emergence of hyperglycemia and failing β-cell function, its value may be limited. Similarly, QUICKI and Matsuda index are also contained in their usefulness with declining β-cell function^29^. Therefore, hyperinsulinemic-euglycemic clamp remains the procedure of choice to assess the whole body insulin sensitivity in patients with T2DM. Hence, we planned to use clamp studies for the assessment of insulin sensitivity in our study patients.

The strategies to target insulin resistance in patients with T2DM include life-style modification, metformin and thiazoledinediones^30^. The latter drug prototype include pioglitazone, which is a selective PPAR-γ agonist and improves insulin sensitivity by augmenting insulin signalling at insulin-target sites, and further by reducing lipotoxicity and proinflammatory adipocytokine, tumor necrosis factor-ά (TNF-ά), promote insulin action^31^.Lipotoxicity is one of the pivotal mechanism implicated in mediation of insulin resistance at insulin target sites as well as β-cell dysfunction. The key metabolites for inducing insulin resistance through lipotoxicity include non-esterified fatty acids and triglycerides. Impaired triglycerides metabolism in the liver and muscle in diabetes results in further production of various noxious metabolites such as fatty acyl conenzyme A, ceramides and diacylglycerol. Lipotoxicity may be attenuated with the use of PPAR-ά agonist, which by reducing triglycerides may attribute to improvement in insulin sensitivity^32^. Fenofibrate, a PPAR-ά agonist is widely used drug in the management of hypertriglyceridemia that effectively reduces serum triglycerides levels and modestly improves insulin sensitivity^33^. Saroglitazar, a predominant PPAR-α and moderate γ-agonist, is recently approved drug for the management of diabetic dyslipidemia and has been demonstrated to decrease triglycerides and improves HbA1c^34^; however, its effect on insulin sensitivity remains unexplored. In our study, saroglitazar significantly improved insulin sensitivity accompanied with significant decrease in HbA1c and TG levels, major attributes to reduction in gluco-lipotoxicity. Further, the effect on SIclamp persisted despite adjusting for weight, HbA1c, TG and HDL-C. However, change in insulin sensitivity in subjects receiving saroglitazar did not correlate significantly with changes in TG and HbA1c levels. Therefore, it is conceivable that beside reduction in gluco-lipotoxicity, it also exerts its additional moderate effect directly through PPAR-γ receptors that stimulates the transcription of several insulin-responsive genes, thereby, further contributing towards the improvement in insulin sensitivity. Alternatively, a small sample size and short duration of the study may have precluded its (saroglitazar) mediation of its beneficial effects through gluco-lipotoxicity alone.

Saroglitazar administration resulted in a significant reduction in FPG and HbA1c as compared to placebo. However, it was not accompanied with increase in fasting plasma insulin and C-peptide indicating that improvement in glycemic profile was possibly contributed by enhanced insulin sensitivity. Further, a significant decrease in FPG itself denotes improved hepatic insulin sensitivity as PPAR-α are predominantly expressed in liver, a major target site for saroglitazar. A significant change in HOMA-β was observed in the saroglitazar group which also showed a trend towards significance to SIclamp again indicating that improvement in β-cell function was a consequence of improved insulin sensitivity.

However, a modest direct effect of saroglitazar through PPAR-γ and α-agonist activity on β-cells cannot be excluded, as both these receptors are abundantly expressed on β-cells^35^. In addition, both the groups were well matched with respect to the median glimepiride dose, hence it seems plausible that the measured difference in HbA1c and FPG is likely ascribable to the effect of saroglitazar itself. While previous trials with saroglitazar^36^ and aleglitazar noted improvement in glycemic parameters, muraglitazar failed to cause a significant change in HbA1c^26^. This differential effect could be due to the variance in relative affinities of these drugs for PPAR-α and PPAR-γ receptors as well as differences in molecular weight and structure^37,38^.

In our study, both the groups had matched total cholesterol, LDL-C and apoB levels at baseline. However, baseline serum TG levels were higher and serum HDL-C levels were lower in patients receiving saroglitazar as compared to those randomized to placebo. The difference in baseline triglyceride existed despite randomization and blinding as the patients were not stratified as per triglyceride or HDL-C levels. Saroglitazar significantly lowered triglycerides and raised HDL-C levels as compared to placebo even after adjusting for the baseline differences. A similar effect on triglyceride and HDL-C levels with negligible effects on LDL-C levels has also been shown with muraglitazar^26^ and aleglitazar^39^, previously. We did not find lowering of LDL-C, apoB and total cholesterol unlike PRESSV, where significant lowering of above parameters were noted with saroglitazar. This discrepancy may be due to a relatively small sample size of our study whereby changes of smaller magnitude may not be easily apparent.

No significant effect of saroglitazar was noted on different anthropometric parameters, including body weight, BMI, waist circumference or body fat%., as measured by DEXA. Similar findings were also noted in PRESS V and PRESS VI^36^. Interestingly, studies with aleglitazar and muraglitazar had shown an increase in body weight along with an increase in fat mass^26,39^ inspite of similar study duration indicating that these may be a drug-specific effect rather than class action.

No adverse effects accountable to saroglitazar use were observed nor were any episodes of symptomatic hypoglycemia reported during the clamp study. The plasma glucose was analysed at 5 minutes interval during the clamp procedure to ensure the rapid correction of hypoglycemia, if noted. This is in accordance with the findings of PRESS V and PRESS VI trials wherein no serious adverse events were reported with saroglitazar^36^.

Our study has a number of strengths. As a pilot study, it was able to demonstrate a clinically and statistically significant improvement in insulin sensitivity as well as HbA1c. The groups were well matched with respect to most metabolic and laboratory parameters. Most patients (25/30, 83.3%) were able to complete the study with no adverse events. Our study had a few limitations. Owing to the invasive and time consuming nature of hyperinsulinemic euglycemic clamp, we recruited a small number of patients. Also, the absence of an active comparator limits the generalizability of the results. Further, we could not explore into mechanistic insights regarding improvement of insulin sensitivity with saroglitazar by estimating non-estified fatty acids and TNF-ά. Expectedly, our results will act as a harbinger for larger trials which can overcome these limitations.

## Conclusion

We conclude that saroglitazar effectively reduces hypertriglyceridemia and improves insulin sensitivity along with β-cell function by reduction in gluco-lipotoxicity and possibly directly through PPAR-γ agonism in patients ofT2DM with hypertriglyceridemia.

## Data Availability

The datasets supporting the conclusions of this work are included in the article.On reasonable request, the content can be available from the corresponding author.
